# Rare Chance for a Doctor

**Published:** 1883-11

**Authors:** C. I. Burroughs

**Affiliations:** Eden, Ga.


Rare Chance for a Doctor.—A practice worth $2,500, with large, comfortable house and office, barn, stables, etc., with 50 acres of land; no competition; good pay. Will sell or rent on easy terms.

Address C. I. Burroughs, M.D., Eden, Ga.

